# Integrating electroactive microorganisms into active soil management strategies

**DOI:** 10.3389/fmicb.2026.1753999

**Published:** 2026-02-11

**Authors:** Lenon Romano Modesto, Ignacio Baquedano, Ezgi Öğün Ramalhete, Silvia Mena, Mukesh Sharma, Pablo Rodríguez-Núñez, Ivana Danilov, Dibyojyoty Nath, Natasha Tait, Ignacio Javier Moro, Uliana Reutina, Işıl Yücel, Snežana Vučetić, Alicia Prieto, David Colliaux, Jorge Barriuso, Gonzalo Guirado, Ioannis Andrea Ieropoulos, Xavier Munoz-Berbel, Jovana Grahovac, Peter Hanappe, Naroa Uria, Markus R. Schmidt, Rachel Armstrong

**Affiliations:** 1 Paris Research, Sony Computer Science Laboratories, Paris, France; 2 Centro de Investigaciones Biologicas Margarita Salas, Madrid, Spain; 3 Katholieke Universiteit Leuven Faculteit Architectuur, Brussels, Belgium; 4 Universitat Autonoma de Barcelona, Barcelona, Spain; 5 University of the West of England, Bristol, United Kingdom; 6 Instituto de Microelectronica de Barcelona, Cerdanyola del Vallès, Spain; 7 Univerzitet u Novom Sadu, Novi Sad, Serbia; 8 School of Engineering, University of Southampton, Southampton, United Kingdom; 9 Arkyne Technologies Bioo, Barcelona, Spain; 10 Biofaction KG, Vienne, Austria

**Keywords:** agriculture, genomics, innovation, MFC, sustainability

## Abstract

Electroactive microorganisms (EAMs) can be incorporated into active soil management as a strategy for regenerative agriculture. Through extracellular electron transfer, they drive nutrient cycling, biofertilization, and pollutant degradation while also producing bioelectricity. Soil microbial fuel cells exemplify their use as self-powered biosensors and platforms for bioremediation. Reframing soils as dynamic bioelectronic interfaces, EAMs enable nutrient recovery, waste valorisation, and resilience. The concept of “gardening microorganisms” integrates them as programmable agents within managed ecosystems. By coupling microbial consortia engineering, bioelectronic scaffolds, and circular nutrient recovery, soils work as intelligent, self-regulating systems. This review positions EAMs as a tool in soil management for shaping climate-smart, regenerative agroecosystems that sustain productivity and ecological balance.

## Introduction

Regenerative agriculture harnesses the power of microorganisms to restore soil health, enhance nutrient cycling, and rebuild organic matter. By fostering microbial diversity through practices like reduced tillage and cover cropping, it creates living soils that support resilient crops, sequester carbon, and regenerate degraded ecosystems (Singh et al., 2023). This approach emerges as a response to the consequences of intensive agricultural practices, which are generating unprecedented environmental pressures that compromise global food security and undermine ecosystem resilience (Hultgren et al., 2025). Such practices have led to widespread soil degradation, depletion of organic matter, and erosion of microbial biodiversity (Kruczyńska et al., 2023; Leul et al., 2023). These converging pressures demand urgent rethinking around agricultural systems, necessitating a shift from extractive models toward regenerative strategies that restore soil health, close nutrient cycles, and reduce dependency on external chemical inputs (Li et al., 2024; Taniushkina et al., 2024). Global reliance on synthetic fertilizers, particularly nitrogen-based products, has increased crop productivity but caused soil damage, nutrient imbalances, and deficiencies (Yilmaz and Yilmaz, 2025), greenhouse gas emissions, and contamination of waterways (Ishfaq et al., 2023). Phosphorus, a vital macronutrient, and key component of phosphate-based fertilizers essential for sustaining high-yield agriculture, is primarily derived from finite phosphate rock reserves, which might peak by 2033 and face complete depletion within the next century (Illakwahhi et al., 2024). In addition, the accelerating impacts of climate change—rising temperatures, altered rainfall patterns, and extreme weather—are compounding soil instability and reducing arable land area (Olsson et al., 2023; Li et al., 2024).

Microorganisms are central to regenerative practices not only as key players in plant holobionts, driving nutrient cycling and resilience, but also as dynamic partners in shaping agroecosystem function (Singh et al., 2023). Moving beyond restoration, their integration through electroactive interfaces positions them as living sensors, off-grid power sources, and effectors, while actively participating in nutrient cycling and supporting the diverse soil microbiota of agricultural systems. Building on this potential, electroactive microorganisms (EAMs) represent an emerging frontier in the transition, as they move beyond supporting microbial communities to being directly integrated into bioelectrochemical systems (BES) that mediate processes such as C and N cycles, where their metabolic versatility can be directed toward ecological regeneration (Ren et al., 2025). These “microbial powerhouses” are capable of extracellular electron transfer (EET), linking their metabolism directly to redox-active compounds and solid-state electrodes (Qin et al., 2024; Ren et al., 2025). Through this unique capability, EAMs can simultaneously oxidize organic matter, drive the release of nutrients, and generate useful levels of electrical output (Qin et al., 2024). Applications such as soil-based microbial fuel cells (SMFC) illustrate this dual potential: by exploiting redox gradients in soil, EAMs can enhance nitrogen cycling, solubilize phosphates and micronutrients, and support plant growth, while also producing signals or currents that can be used for sensing and energy recovery (Gao et al., 2024). In addition, EAMs are increasingly recognized for their contributions to biogeochemical cycling, rhizosphere interactions, biosensing, and even novel material production, positioning them as key agents in the design of closed-loop, low-impact agricultural frameworks (Gao et al., 2024; Nath and Ieropoulos, 2025).

This article synthesizes current knowledge on the ecological and biotechnological potential of EAMs within active soil management strategies. We first outline the core microbial genera and functional traits that underpin EET, with emphasis on their relevance to nutrient cycling, biofilm formation, and rhizosphere interactions. We then discuss the state of the art for identifying and designing MFC tailored microbial consortia. Finally, we highlight the application pathways of EAM-based systems in regenerative agriculture, including biofertilization, soil sensing and remediation, and novel bioproducts. By situating EAMs within the broader context of soil heath dependency, and climate-driven challenges, this review frames them not as microbial curiosities but as emerging powerhouses with the capacity to transform agroecosystems. The objective of this article is to define and critically examine a framework for integrating electroactive microorganisms (EAMs) into soil–plant systems. In doing so, it identifies key knowledge gaps and research priorities required to advance EAM-based soil systems from proof-of-concept studies toward reliable, field-relevant applications.

## Electromicrobiology

Electromicrobiology is an emerging sub-discipline of microbiology that studies microorganisms capable of EET to or from insoluble metal ions or solid surfaces acting as electron acceptors or donors (Lovley, 2012; Nealson and Rowe, 2016). This process represents a novel form of respiration that enables energy production and cellular activity in environments where dissolved oxygen is scarce and soluble terminal electron acceptors (TEA) such as nitrate and sulfate are limited (Rabaey et al., 2007). Central to this process are cytochromes, heme-proteins that evolved to incorporate iron into their structure, allowing rapid electron transfer from the cytoplasm to external acceptors via iron-associated proteins (Jiang et al., 2020). Similar copper- and manganese-binding protein complexes suggest convergent evolutionary solutions for EET across microbial lineages. These electroactive traits are widespread across the three domains of life, leading to the adoption of the term “electroactive microorganisms (EAM)” (Logan, 2009; Logan et al., 2019). The first EAMs were isolated by Derek Lovley from Potomac River sediments, including *Geobacter metallireducens* and *Geobacter sulfurreducens*, which displayed dissimilatory (energy-generating rather than biomass-forming) metal-reducing activity (Lovley and Phillips, 1988; Lovley et al., 1989). *G. metallireducens* can reduce metals such as Fe(III), Mn(IV), and U(VI), coupled with the oxidation of organic carbon sources like acetate, glucose, and fumarate (Lovley et al., 2011). Since then, more than 100 novel EAM strains have been identified, confirming that electroactivity is widespread among bacteria, archaea, and eukaryotes, and highlighting the ecological importance and biotechnological potential of electromicrobiology (Schröder and Harnisch, 2017; Logan et al., 2019). EAMs include bacteria such as *Geobacter metallireducens*, *Geobacter sulfurreducens*, *Shewanella oneidensis* MR-1, *Escherichia coli*, *Pseudomonas aeruginosa*, *Bacillus subtilis*, and *Klebsiella aerogenes*. They also encompass archaeal taxa, as methanogenic lineages such as *Methanospirillum hungatei* and *Methanosarcina barkeri*, as well as anammox-associated bacterial groups, including *Candidatus Brocadia* sinica and *Candidatus Scalindua* (Logan et al., 2019; Walker et al., 2019; Yu et al., 2020). In addition, eukaryotes, including *Saccharomyces cerevisiae* and fungi like *Candida melibiosica*, can generate electricity in microbial fuel cells (MFC) (Logan et al., 2019). A striking example is cable bacteria, which form multicellular filaments capable of transporting electrons over distances greater than 1 cm (Logan et al., 2019). *Shewanella oneidensis* MR-1, discovered by Kenneth H. Nealson, was named after its genus (*Shewanella*), site of isolation (Oneida Lake, New York, United States), and its dissimilatory manganese-reducing property (“MR-1”) (Venkateswaran et al., 1999; Nealson et al., 2003). This strain, along with other microorganisms such as the cyanobacterium *Synechocystis* PCC6803 and the fermentative bacterium *Pelotomaculum thermopropionicum*, can also produce electrically conductive nanowires (Gorby et al., 2006).

EAMs are further categorized by their electricity production in MFCs based on power density (PD) (Doyle and Marsili, 2018; Logan et al., 2019):

(1) Poor EAMs: PD (<10 mW m^−2^), EET is not efficiently carried out.
(2) Good EAMs: PD (>10 mW m^−2^ and <100 mW m^−2^). These microbes can be considered as standard EAMs.
(3) Very good EAMs: PD (>100 mW m^−2^) is calculated under optimal configurations and other operating conditions in MFC.
(4) Excellent EAMs: PD (>1,000 mW m^−2^).

## Long-distance electron transfer and electroactive biofilms

EAMs have developed specialized mechanisms to exchange electrons with external electron acceptors and donors across remarkable spatial scales. Investigations in marine sediments revealed that microbial activity in anoxic sulfide layers was coupled with oxygen reduction in oxic layers several centimeters apart (Nielsen et al., 2010). This phenomenon was attributed to long-distance electron transfer (LDET), initially assumed to occur via conductive minerals such as humic substances or magnetite. However, subsequent work identified conductive “cable bacteria” that transfer electrons over centimeter distances — far exceeding the nanometer–micrometer range of mineral-based conductivity (Müller et al., 2016; Meysman, 2018). Similar mechanisms are observed in *Geobacter* and *Shewanella oneidensis*, which use Type IV pili and nanowires, with recent discoveries of highly conductive OmcZ nanowires exhibiting rates 1,000-fold greater than OmcS (Snider et al., 2012; Yalcin et al., 2020). These strategies allow microbes as small as 2 μm to achieve electron transfer across 1–2 cm, a scale 10^3^–10^6^ times longer than their body length (Li et al., 2016). This capacity for LDET acts as a microbial communication and survival strategy, comparable to long-range “telephonic conversations” that allow distant communities to coordinate metabolic activities (Pirbadian and El-Naggar, 2012; Jiang et al., 2020). Such processes are critical in biogeochemical cycling, bioremediation, and soil ecology, where microbial electron flow influences redox gradients and resource distribution (Pfeffer et al., 2012; Schauer et al., 2014).

A major ecological manifestation of this phenomenon is the formation of electroactive biofilms. In direct electron transfer pathways, microorganisms irreversibly attach to solid surfaces such as electrodes, producing stratified biofilms (Kiran and Patil, 2019). These biofilms establish gradients of substrates and terminal electron acceptors, where outer-layer cells access nutrients while inner-layer cells face starvation, leading to inactive layers and detachment (Greenman et al., 2021a). Nonetheless, electroactive biofilms are uniquely resilient, sustaining EET under extreme pH, temperature, pressure, or radiation. By facilitating LDET and robust community structures, these biofilms underpin applications ranging from methane mitigation and wastewater treatment to soil restoration and rhizosphere management. Together, the discovery of cable bacteria and conductive nanowires, alongside the ecological organization of electroactive biofilms, highlight how EAMs have converged on structural and community-level strategies to extend the spatial reach of electron flow and thrive in dynamic, often harsh, environments.

## The biology and ecological relevance of electroactive microorganisms

**Figure 1:**
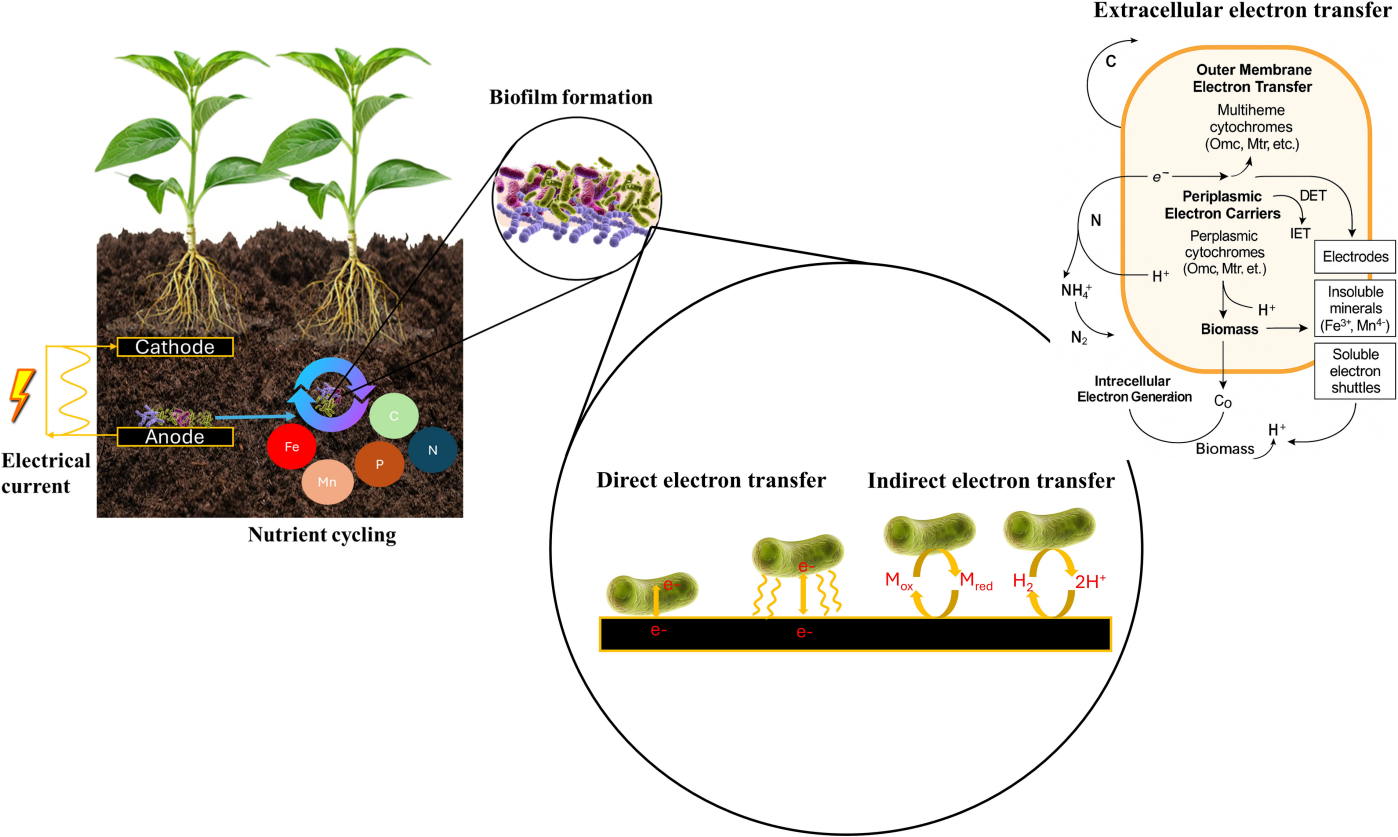
Electroactive microorganisms in soil: electrical current generation, nutrient cycling, biofilm formation, and extracellular electron transfer pathways integrated with carbon and nitrogen cycles. Intracellular oxidation of organic substrates produces electrons (e^−^), protons (H^+^), and CO_2_. Electrons are transferred through periplasmic and outer membrane cytochromes and nanowires to external acceptors via direct electron transfer (DET) through conductive pili or indirect electron transfer (IET) mediated by soluble redox shuttles. In IET, mediators alternate between oxidized (M_ox_) and reduced (M_red_) states (M_ox_ ↔ M_red_), shuttling electrons between cells and external acceptors. Electrons may also reduce protons (2H^+^ + 2e^−^ → H_2_), linking microbial metabolism with hydrogen evolution. Protons are released into the medium to maintain redox balance. This process is linked to the carbon cycle through CO_2_ release and microbial biomass assimilation, and to the nitrogen cycle through transformations of NH_4_^+^, NO_3_^−^, and N_2_ via nitrification and denitrification.

Plants and their associated microbiomes are closely linked to the activity of EAMs, particularly in plant–MFCs. In these bioelectrochemical systems, plant roots release organic exudates into the rhizosphere, which serve as substrates for EAMs. By oxidizing these compounds, EAMs generate electrons that are transferred to electrodes through EET, thereby coupling plant growth with bioelectricity production (Logan et al., 2019). This demonstrates that EAMs are not only microbial specialists but can also be integral partners in plant–soil–electrode interactions (Figure 1).

EAMs perform EET to exchange electrons with external minerals, electrodes, or other microbes (Shi et al., 2016; Doyle and Marsili, 2015). EET is typically divided into two mechanisms: direct electron transfer (DET), mediated by conductive pili (nanowires), biofilms and outer-membrane cytochromes (Reguera, 2012; Malvankar et al., 2015), and indirect electron transfer (IET), which relies on soluble redox-active mediators (Chiranjeevi et al., 2019; Gemünde et al., 2022) (Figure 1). In *Shewanella* and *Geobacter*, DET is facilitated by conductive pili and multi-heme cytochromes, including the Mtr/Omc pathways (Pirbadian et al., 2014; Lovley, 2017). In *Shewanella oneidensis* MR-1, proteins such as CymA, MtrA, MtrB, MtrC, and OmcA bridge the quinone/quinol pool in the cytoplasmic membrane to outer-membrane receptors, enabling electron transfer to Fe-containing mineral surfaces. These pili and cytochromes function as nanowires that enhance DET efficiency by anchoring bacteria to distant electron acceptors (Gorby et al., 2006). In contrast, IET depends on electron shuttles, which may be endogenous (e.g., flavins such as riboflavin and FAD) or exogenous (e.g., AQDS, neutral red, methylene blue) (Gralnick and Bond, 2023). Some bacteria, such as *Pseudomonas aeruginosa*, produce self-secreted mediators like pyocyanin, while synthetic mediators can be added to enhance electron recovery (Gemünde et al., 2022; Arbour et al., 2020). Another defining feature of EAMs is their metabolic versatility. They can couple EET to a wide range of catabolic and anabolic pathways, using soluble substrates (e.g., organic acids, hydrogen), insoluble donors or acceptors [e.g., Fe(III) oxides] (Richardson et al., 2012), and even synthetic electrodes (Wang et al., 2022; Gemünde et al., 2022). This flexibility enables them to adapt to dynamic environments where conventional respiration is not viable.

The ecological relevance of EAMs is reflected in their contributions to global biogeochemical cycles. In the carbon cycle, they degrade organic matter and reduce CO₂ into biomass or intermediates via pathways such as the reductive TCA cycle or the Wood–Ljungdahl pathway, while coupling electron release to the reduction of insoluble metal oxides or electrodes, thereby influencing carbon fluxes in soils and sediments (Cai et al., 2022; Yang G. et al., 2025). During anaerobic respiration, EAMs oxidize substrates such as acetate or lactate, coupling electron release to the reduction of metal oxides or electrodes, and redirect electrons toward reductive pathways, including autotrophic CO₂ fixation, highlighting their metabolic versatility at the interface of the carbon cycle and redox-active environments (Lovley, 2017; Igarashi and Kato, 2017; Nevin et al., 2010; Rabaey and Rozendal, 2010). In the nitrogen cycle, EAMs mediate nitrate reduction and denitrification through canonical reductases encoded by nar, nir, nor, and nos gene clusters, coupling extracellular electron uptake to the production of ammonium or dinitrogen under anoxic conditions. In agricultural systems, these processes are complemented by symbiotic nitrogen-fixing microbes such as *Bradyrhizobium* spp. that provide ammonium to plants (Kuypers et al., 2018; Li et al., 2025).

Sulfur and iron transformations further exemplify the coupling between EET and biogeochemical processes. In sulfur cycling, assimilatory and dissimilatory sulfate reduction pathways involving ATP sulfurylase, APS reductase, and dissimilatory sulfite reductase (dsrAB) intersect with denitrification and are functionally connected to extracellular electron flow (Slobodkin and Slobodkina, 2019; Qian et al., 2024; Zhuang et al., 2024; van Vliet, 2021). In the iron cycle, outer-membrane cytochrome complexes and conductive pili enable the reduction of insoluble Fe(III) minerals, producing secondary Fe(II)-bearing phases that can be reoxidized by iron-oxidizing microorganisms (Igarashi and Kato, 2017; Zhang et al., 2023a; González-Paz et al., 2022). Mechanistic insights from model organisms such as *Shewanella* and *Geobacter* illustrate conserved molecular strategies for EET across diverse taxa (Shi et al., 2016; Lovley, 2017). Collectively, these gene-encoded pathways demonstrate how EAMs couple intracellular metabolism to extracellular redox reactions, linking molecular-scale electron transfer to ecosystem-level biogeochemical cycling in both natural and engineered environments.

## EAM design and monitoring

Microbial communities colonizing the anode of MFCs play a crucial role by simultaneously metabolizing compounds, transferring electrons to the electrode, and removing pollutants from the environment (Yin et al., 2025; Ahirwar et al., 2025). These communities can also be harnessed for producing metabolites such as vitamins, organic acids, and phytohormones with applications in hydroponics, effectively functioning as a prosthetic rhizosphere that substitutes for plant root microbiomes (Sato et al., 2023; Nath and Ieropoulos, 2025). Rationally designing these microbial consortia offers opportunities to improve power output and provide additional functions beneficial to plants, including phosphorus solubilization and the production of hormones or siderophores.

Two complementary strategies exist for MFC community design. Top-down approaches rely on naturally diverse microbial communities capable of both degrading organic matter and performing extracellular electron transfer. Activated sludge from wastewater treatment plants is the most common inoculum, but sediments, digester effluents, compost leachates, and agricultural soils and residues are also used (Gu et al., 2024; Sun et al., 2024). Fungi frequently occur in these consortia and contribute to plant biomass-derived polymers degradation (Toczyłowska-Mamińska et al., 2020). Community performance can be enhanced through bioaugmentation with specific strains to reinforce pollutant degradation or electron transfer, while advanced sequencing (e.g., genomics and metagenomics) reveals taxonomic composition and functional potential (Nachammai et al., 2023; Harada et al., 2025). These techniques, such as shotgun or 16S or 18S rDNA amplicon sequencing, can be used to monitor changes induced in EAM communities through bioaugmentation processes. On the other hand, bottom-up approaches, in contrast, assemble defined consortia of microorganisms with targeted functions, such as fermenters, electroactive bacteria, or biosynthesis specialists. This strategy allows increased energy production, remediation, or biosynthesis functions, though it requires detailed understanding of interspecies interactions to maintain stable performance (Zhang J. et al., 2025). To gain a deeper understanding of the microorganisms that constitute the artificial community, it is essential to analyze their metabolic potential. This can be accomplished using genomic and metabolomic approaches, which reveal the functional capabilities of the organisms.

Community efficiency at the anode is determined by trophic, physical, and signaling factors (Uria et al., 2017). Trophic interactions create syntrophic chains where degraders hydrolyse complex organics into intermediates like acetate or lactate, which are then used by electroactive species. These interactions can be modulated by introducing specific microbial strains or through metabolic engineering. Physical factors include the development of stable conductive biofilms embedded in extracellular polymeric substances (EPS), which enable close electrode contact and efficient electron transfer. Biofilm architecture influences diffusion and electrochemical activity and can be studied using Scanning electron microscope (SEM), confocal laser scanning microscope (CLSM), or flow cytometry (Catal et al., 2024; Li F. et al., 2023; Lin et al., 2024). Signaling molecules, including quorum-sensing autoinducers, regulate biofilm growth, EPS production, and EET-related gene expression, orchestrating cooperation and stability within anodic communities (Chen et al., 2017; Wu et al., 2022).

To deepen understanding, multi-omics and modeling approaches are increasingly used. Metatranscriptomics identifies actively expressed genes, while metabolomics reveals exchanged intermediates and signaling compounds (Nachammai et al., 2023; Castellano-Hinojosa et al., 2025). Computational models integrate these insights, clarifying which parameters most affect performance and predicting community behavior under varying conditions. Models range from differential equation frameworks that combine biological dynamics with electrochemical kinetics (Picioreanu et al., 2007) to spatially explicit approaches such as agent-based models, which capture biofilm structure, quorum sensing, and interspecies interactions (Wang J. et al., 2023; Wang et al., 2025). Biofilm simulations can be performed with dedicated software (Cockx et al., 2024; Breitwieser et al., 2023), and artificial neural networks have also been applied to predict MFC performance (Karamzadeh et al., 2023; Potrykus et al., 2025). Together, these design and monitoring strategies provide the foundation for developing MFC systems that are not only efficient in energy generation but also adaptable for environmental and agricultural applications.

## EAMs in soil and their significance

EAMs access the Earth’s power grid—natural redox-active minerals and electron flows in soils and sediments—through their long conductive nanowires, Type IV pili, or directly through cell surface proteins or self-secreted electron shuttles for their survival, energy production, and interspecies communication. For example, *Geobacter* and *Shewanella* species are strict anaerobes; hence, for energy production (adenosine triphosphate or ATP), they break down the chemical bonds present in organic compounds and transfer the free electrons to an electron-deficient/withdrawing matter present at the cell exterior through EET. In anaerobic natural habitats, a wide variety of electron acceptors are abundantly available, such as Fe(III), Mn(VI), NO₃^−^, and SO₄^2−^, or between syntrophic partners (such as *Geobacter* with methanogens or sulfate-reducing bacteria) and other non-mutualistic interactions (host–microbes) that act as TEA (Nealson et al., 2003; Schröder and Harnisch, 2017; Moscoviz et al., 2020). These conductive structures effectively function as plugs that connect microbes into a natural “electrical grid” in the soil, a system that may be responsible for allowing many types of microbes to survive and support life (Portela et al., 2024). Importantly, this EET-based respiration enables EAMs to live in some of the harshest conditions on Earth, including acid-drainage mines, undersea vents, and anaerobic environments with limited soluble TEAs (Logan et al., 2019; Moscoviz et al., 2020). The EET also provides EAMs with the ability to enrich the surface of solid copper electrodes and to reduce toxic metals such as Cr(VI) and U(VI) (Lovley et al., 1991; Beuth et al., 2020; Yuan et al., 2020). The biological respiration that proceeds from electron donors to a final electron acceptor is spontaneously carried out in natural soils and sediments through potential differences between two electrochemical gradients. For example, in anaerobic environments, microorganisms respire on soluble metals such as Fe(III) and Mn(IV), or other electron acceptors like NO₃^−^, SO₄^2−^, or CO₂ to form CH₄ (methanogenesis), or they may follow fermentative pathways for energy production. In contrast, oxygen in the aerobic zone acts as the TEA. As a result, a thermodynamic disequilibrium develops between these zones, where multiple gradients drive the flow of electrons from electron-rich (negative) to electron-deficient (positive) areas, effectively forming a virtual natural biofuel cell in soils and water bodies (Schröder and Harnisch, 2017). Because of these capabilities, EAMs play a key role in the biogeological cycle and natural bioremediation processes on the Earth’s surface (Beuth et al., 2020; Yuan et al., 2020), hence they could be called “Guardians of the Environment.” However, despite years of research, there are no direct measurements showing what percentage of soil biogeochemical cycling is driven by EAM versus other microbial groups, or what proportion of total soil electron flux moves through electroactive pathways. Researchers can distinguish healthy from unhealthy soil using electrochemical signals from EAB, but cannot quantify their actual functional contribution to ecosystem services like nutrient cycling or carbon sequestration (Mohamed et al., 2021). Functional genes involved in carbon, nitrogen, and phosphorus cycling explained biogeochemical process variation based on soil stoichiometry, but again, this does not identify EAM specifically (Trivedi et al., 2016).

## Soil microbial fuel cells: enabling technologies and ecological impact

SMFCs exploit natural redox gradients in soils to enable EAMs to oxidize organic substrates, generating electricity while driving nutrient transformations (Das and Mishra, 2025; Taylor et al., 2024). These systems rely on native microbial communities and soil matrices to promote redox reactions, producing power that can support applications such as sensors or irrigation controllers (Ancona et al., 2020; Nakamoto et al., 2024). Typically, the anode is buried in anaerobic soil layers where electroactive biofilms form, while the cathode is placed closer to the surface where oxygen serves as the final electron acceptor, creating a spatial redox interface that sustains both current generation and nutrient cycling (Garbini et al., 2023). Soil physical and chemical properties, including water content, texture, and organic matter, strongly influence SMFC performance (Yen et al., 2024). High water levels improve ion transport and reduce internal resistance, while soil structure and amendments such as biochar regulate oxygen diffusion and redox heterogeneity (Joseph et al., 2015; Lacroix et al., 2023). These conditions create microscale redox niches within soil aggregates or the rhizosphere, where microorganisms adapt to different electron acceptors ranging from oxygen in well-aerated soils to nitrate, Fe(III), Mn(IV), sulfate, or CO₂ under waterlogged or anaerobic conditions (Marschner, 2021; Bhattacharyya et al., 2018; Kuleshova et al., 2022). For pollutants like organic matter and heavy metals, nutrient removal efficiencies in laboratory systems frequently exceed 90% (Golzarian et al., 2024; Niu et al., 2025). Remediation in the field faces additional challenges, such as ensuring that treatment is not confined to areas near electrodes and managing longer timeframes (from days to several weeks) to achieve similar efficiencies (Fatehbasharzad et al., 2022; Lu et al., 2025).

In SMFCs, microbial metabolism couples current generation with nutrient transformation. Denitrification by EAMs reduces nitrate loads, while phosphorus is mobilized from insoluble minerals into bioavailable forms, and Fe(III) and Mn(IV) are reduced into more soluble, less toxic species (Garbini et al., 2023; Ojha et al., 2025). These processes enhance nutrient recovery, including compounds such as vivianite and ammonium, and depend on electrochemical gradients driven by current density (Li et al., 2020). By spatially separating redox processes, SMFCs release organic matter, stimulate nutrient mineralization, and improve soil fertility, particularly in agricultural soils where nutrient availability limits crop productivity (Bhowmik et al., 2017; Bayu, 2024). The integration of SMFCs into regenerative agriculture offers pathways to reduce dependence on synthetic fertilizers by enhancing natural nitrogen and phosphorus cycling, while supporting microbial diversity that underpins soil resilience (Umar et al., 2023). As a result, SMFCs transform soil into an active bioelectrochemical system that regenerates fertility and sustains crops with lower external inputs. Recent studies show SMFCs can also improve soil biochemical properties, including higher ATP concentrations and greater ammonium availability from mineralized organic matter, boosting productivity in organic farming systems (Chong et al., 2025). Beyond soil health, they provide self-sustaining, low-energy power for monitoring and precision agriculture tools, reinforcing their dual role as both ecological enhancers and enabling technologies.

## From soil sensors to rhizosphere interfaces: applied innovations

Beyond renewable energy, wastewater treatment, and hydrogen production, the use of MFCs as biosensors has gained attention due to their low cost, robustness, and ability to self-power (Arun et al., 2024). Acting simultaneously as biological recognition elements and transducers, MFC sensors can detect analytes and monitor environmental quality in water (Olias and Di Lorenzo, 2021), air (Jiang et al., 2018), and soils (Kilinc and Catal, 2023) in real time. Heavy metals such as Hg^2+^, Cd^2+^, Cr^2+^, Zn^2+^, Cu^2+^, Pb^2+^, and Ni^2+^ inhibit electroactive microbial metabolism, reducing current output and serving as toxicity indicators (Naik and Jujjavarapu, 2021; Zhang et al., 2022; Adekunle et al., 2023). Wang et al. demonstrated stable detection of multiple metals (Cd^2+^, Zn^2+^, Pb^2+^, Hg^2+^) over four months using a carbon felt cathodic SMFC biosensor enriched with *Pseudomonas*, *Geobacter*, and *Desulfobulbus* species (Wang S. H. et al., 2023). Other environmental parameters, including pH and temperature, also correlate with electrochemical signals, as acidity inhibits enzymatic activity (Lim et al., 2022), while temperature linearly enhances power output (Greenman et al., 2021b). MFC biosensors have also been applied for volatile fatty acids (Sun et al., 2021) and biological oxygen demand (BOD), with detection ranges up to 1,280 mg/L depending on design (Do et al., 2020; Wang et al., 2018; Wang S. H. et al., 2023).

In addition to sensing, MFCs can harvest and supply energy for low-power applications. Following a lag phase for biofilm formation, electroactive bacteria support autonomous operation of temperature or chemical sensors in both laboratory and field settings (Leicester et al., 2023). Although MFCs produce relatively low voltages (0.5–0.6 V OCV; 0.1–2.0 W m^−2^), power management systems and voltage converters enable their integration into wireless sensor networks, supporting long-term monitoring in remote or resource-limited environments (Nguyen et al., 2019; Kurniawan et al., 2022).

Beyond their technological role, MFC-associated microbes actively enhance soil and water health. Electroactive microorganisms utilize root exudates as electron donors, driving nutrient cycling and redox-mediated transformations such as Fe(III) → Fe(II) reduction (Alzate Zuluaga et al., 2024) and copper removal, with efficiencies of 43–87% while producing approximately 0.5 V electricity (Kavaleuskaya et al., 2025). Hydrocarbons such as phenanthrene have also been efficiently degraded, with removal efficiencies reaching 93.8% in sediment-based MFCs (Hamdan and Salam, 2023). MFC microbiomes further influence plant health by modulating immune responses. Beneficial microbes, including *Pseudomonas* spp., suppress or activate root immunity through pH modulation, hormone regulation, or metabolite secretion (Yu et al., 2019; Li et al., 2021). In *Arabidopsis thaliana*, commensal bacteria activated host genes overlapping with pathogen-induced responses, highlighting their dual role in immune evasion and activation (Hacquard et al., 2017). Electroactive biofilms in the rhizosphere can form conductive networks that reinforce beneficial symbioses, biocontrol activity, and pathogen resistance through sustained metabolite exchange and redox interactions (Li et al., 2021).

At the same time, the deployment of MFCs as rhizosphere interfaces requires careful consideration of ecological risks, trade-offs, and governance. Introducing engineered or highly enriched electroactive microbial consortia may alter native soil microbiomes through competitive exclusion or functional displacement, particularly when non-native or strongly selected strains are used (Fierer, 2017; Van Der Heijden et al., 2008), and may increase the likelihood of horizontal gene transfer in the case of engineered communities (Smets and Barkay, 2005). Recent empirical evidence underscores the magnitude of these concerns. In a controlled study examining the impact of electroactive microbes on farmland soil, locally derived consortia—when enriched in bioelectrochemical systems and re-inoculated at laboratory-optimized densities (OD₆₀₀ 0.8)—induced substantial disruption, with total microbial species diversity declining by approximately 30% and concurrent alterations in soil biochemical properties including pH, total organic carbon, and total nitrogen (Zhang et al., 2023a,b). Critically, this study not only demonstrates the ecological risks of exogenous enrichment, but also reveals it to be an impractical choice because of its high energy and capital demands. To mitigate these risks, current soil-based MFC approaches increasingly favor the enrichment of indigenous electroactive communities rather than exogenous inocula or genetically engineered microorganisms, preserving local microbial diversity and enhancing electrochemical functionality (Doyle and Marsili, 2015). Specifically, this enrichment is achieved through subtle in-situ environmental manipulations rather than exogenous culture additions. These methods include direct electrode deployment for gradual native biofilm colonization, carbon/nitrogen nutrient amendments (compost or biochar addition), and enhancement via plant root exudation—rather than bolus inoculation of concentrated microbial cultures. Empirical evidence from optimized terrestrial MFC systems demonstrates that this in-situ enrichment strategy preserves local microbial diversity without community disruption, in marked contrast to exogenous enrichment approaches (Simeon et al., 2022; Gan et al., 2024). This strategy also reduces biosafety concerns associated with synthetic genetic constructs and the use of selectable markers (Rovira-Alsina et al., 2024). In addition, spatial confinement of biofilms on electrodes or within semi-permeable membranes limits microbial dispersal, while maintaining electrochemical connectivity (Santoro et al., 2017).

From a governance perspective, MFC-enabled soil systems align with emerging frameworks for nature-based solutions and precision agriculture when implemented with transparency, monitoring, and regulatory oversight. Framing soils as bioelectronic interfaces does not imply unrestricted microbial engineering, but rather controlled and measurable interventions that integrate biosafety principles, circular nutrient recovery, and long-term ecosystem resilience. Accordingly, the responsible scaling of electroactive microorganisms-based technologies should be evaluated within existing soil protection, biosafety, and environmental monitoring policies.

## Outlook: EAMs included in the of concept of “gardening microorganisms” in soil

Integrating electroactive microorganisms into active soil management strategies provides a forward-looking framework for designing responsive, self-regulating, and intervention-ready agroecosystems. Building on the foundational science of EAMs, leveraging these organisms as programmable agents within actively managed ecosystems, rather than passive components, can play a useful role in soil management. This proactive “gardening” approach is defined by the active cultivation and real-time stewardship of EAM consortia through bioelectrochemical interfaces to direct soil processes. It differs from general soil health management by its targeted use of specific, engineered microbial functions, and from traditional microbiome engineering by its reliance on continuous in-situ electrochemical feedback to guide microbial activity. This transition from reactive management to microbial “gardening” marks a break from conventional approaches that only address degradation, nutrient loss, and contamination once they become visible (Huang et al., 2025; Garg et al., 2025). Current remediation methods, such as fallowing or nitrate fertilization, remain largely corrective rather than preventive (Govindasamy et al., 2023).

Emerging work on electrotropism and electrical fields demonstrates EAMs influence on plant–microbe interactions (Oikonomou et al., 2024). The integration of bioelectronic substrates, such as cellulose-based conductive polymers (eSoil), offers opportunities for anticipatory and interventionist soil management (Kiprotich et al., 2025). In this context, EAMs can function as both sensors and effectors, detecting early indicators of soil stress and responding through bioelectrochemical signals. The operational “programmability” of these systems can be achieved through specific means: tuning electrode potentials to steer metabolic pathways (e.g., towards nitrogen fixation or metal reduction), using signal molecules to regulate biofilm development, and employing the electrical output itself as a feedback signal for adaptive management. By embedding scaffolds for EAM activity on electrodes or conductive matrices, real-time monitoring of redox potential, pH, and microbial diversity becomes feasible (Chen et al., 2024). This vision must consider the inherent energy constraints of bioelectrochemical platforms, such as the low power output of MFCs for continuous or large-scale soil management operations, necessitating realistic assessments of scalability and long-term deployment (Kurniawan et al., 2022). A pragmatic path to scalability involves hybrid systems where low-power EAM networks function as distributed sensor arrays, rather than primary energy sources, within larger farms. Their cost–benefit must be evaluated against long-term value: reducing input costs via precision biofertilization, averting yield loss through early stress detection, and regenerating soil capital. Key feasibility challenges include engineering robust, low-maintenance electrodes and managing the functional stability of introduced consortia amidst complex soil microbiomes and fluctuating environmental conditions. In addition, translating the capabilities into field-scale systems requires acknowledging the heterogeneity of soil environments—variability in water content, porosity, and texture can significantly affect electron transfer efficiency and metabolic stability, challenging the reliable performance of EAMs beyond controlled settings (Kilinc and Catal, 2023; Wang S. H. et al., 2023). The need of sufficient moisture levels within the reactors, particularly when installed directly in soil, often requires wetland-type or permanently wet soils which reduces the number of suitable locations and increases reliance on the environment (Sakai et al., 2022). Another challenge is the high cost of electrode materials and the fact that long-term operation still requires non-negligible maintenance and monitoring (Borja-Maldonado and López Zavala, 2022).

**Figure 2:**
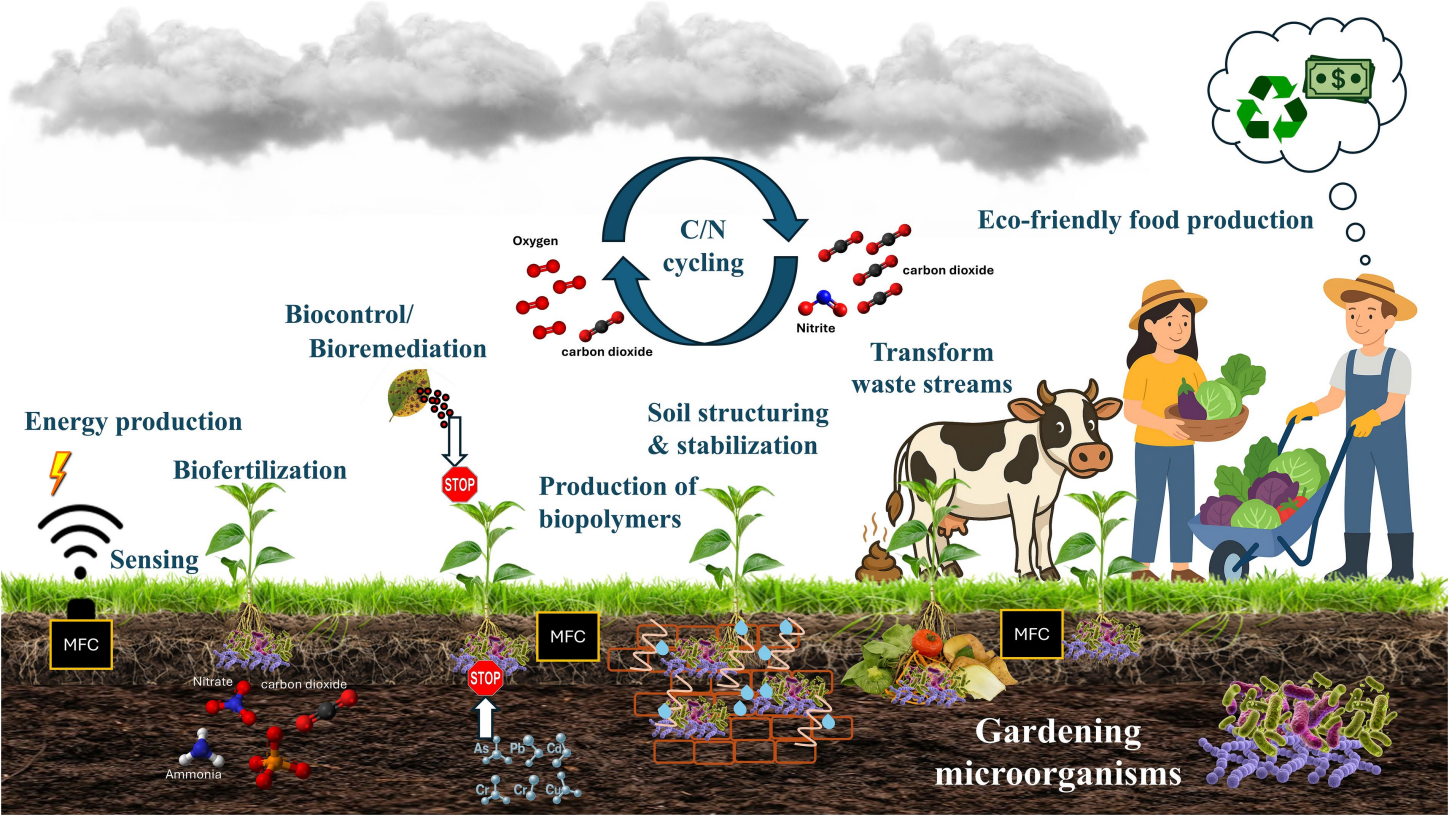
Gardening microorganisms for sustainable systems: microorganisms are cultivated and harnessed for microbial fuel cell (MFC)-based energy production and sensing, while also contributing to plant biocontrol, bioremediation, and soil structuring. Through their metabolic versatility, microbes transform waste streams, drive carbon and nitrogen (C/N) cycling, and produce valuable biopolymers. These integrated microbial processes enhance ecosystem resilience and support eco-friendly food production.

Our vision of “gardening microorganisms” for sustainable systems is illustrated in Figure 2, where microorganisms are cultivated and harnessed not only for MFC-based energy production and sensing, but also for biocontrol, bioremediation, and soil structuring. Their metabolic versatility enables the transformation of waste streams, the regulation of carbon and nitrogen cycling, and the synthesis of valuable biopolymers. Importantly, these EAM-driven functions complement non-electrified agroecological practices—such as reduced tillage, cover cropping, and organic residue retention—which also promote stable microbial habitats, enhance soil organic matter, and support redox-active microbial communities. By aligning EAM activity with these established soil-health strategies, bioelectrochemical approaches can reinforce broader regenerative frameworks rather than functioning as isolated technological interventions. Together, these interconnected microbial functions enhance ecosystem resilience while supporting eco-friendly and regenerative food production.

Although the potential to impact final crop yields and quality is sometimes unclear and remains poorly quantified, EAM have significant potential for targeted biofertilization and biocontrol applications. They are known to link organic matter decomposition with enhanced mineralization, and thus might be used to facilitate nutrient recovery in circular agriculture systems (Qin et al., 2024); however, robust field-scale evidence directly linking these processes to consistent yield or quality improvements is still limited. They can also electro-stimulate nitrogen fixation (Rago et al., 2019), release micronutrients through metal oxide reduction (Zhang et al., 2023b; Zhang et al., 2024), help phosphate solubilization, and enhance siderophore production to improve iron availability while suppressing pathogens (Lozano-González et al., 2023). Despite these promising mechanisms, emerging approaches such as electro-stimulated nitrogen fixation and nanofertilizer production remain largely validated under controlled or laboratory conditions, with few studies reporting variable performance, scalability constraints, or neutral outcomes under agronomic field conditions. Nanoparticle synthesis by *Shewanella* strains provides innovative nanofertilizers or biocontrol agents (Zhuravliova et al., 2023), but concerns related to stability, dose control, environmental fate, and regulatory acceptance still limit their immediate agricultural deployment, while engineered diazotrophic strains such as *Geobacter sulfurreducens* show promise for sustainable nitrogen fixation (Yang W. et al., 2025). Nutrient recovery from microbial electrochemical systems, such as ammonium and phosphate, offers biofertilizer alternatives that reduce reliance on industrial Haber–Bosch synthesis (Zhang X. et al., 2025; Sarangi et al., 2024), although long-term performance, economic viability, and consistency across cropping systems require further validation.

In parallel, EAMs show strong potential for bioremediation applications, reducing toxic metals and degrading pesticides or hydrocarbons (Li K. et al., 2023; Liu et al., 2025). *Pseudomonas* spp. can metabolize persistent pesticides (Książek-Trela et al., 2025), while *Geobacter* species facilitate heavy metal reduction (Silveira et al., 2025). Beyond these single function applications, recent studies highlight the combination of EAM based soil bioremediation technologies with complementary techniques can generate significant synergistic effects. For example in phyto microbial electrochemical systems (PMES), plant exudates stimulate EAM growth while EAMs maintain redox balance, this improves the total petroleum hydrocarbon (TPH) remediation rate significantly compared to phytoremediation alone (Zhang et al., 2021). Amending soil with the biochar is another way of integration to EAM based soil microbial fuel cells to enhance charge generation and increase TPH removal in the anode zone relative to non-biochar amended systems (Rushimisha et al., 2023). Another EAM synergy involves heavy metal passivation enhanced by electrode-driven migration. EAMs like *Acidithiobacillus ferrooxidans* reduce toxic Cr(VI) to immobile Cr(III) while creating acidic conditions (pH 2.0) that promote humic substance formation for metal chelation (Cui et al., 2025). The applied electric field then concentrates these passivated metal complexes near electrodes for easier removal—achieving >50% higher Cr removal than abiotic controls (Yilahamu et al., 2025). Together, these examples illustrate how EAM-based systems can be effectively combined with related technologies to create hybrid technologies. Microbially induced calcite precipitation (MICP) offers another remediation pathway, precipitating pollutants such as As, Pb, Cd, Cr, and Cu as stable carbonates (Comadran-Casas et al., 2025; Zhu et al., 2024; Qin et al., 2025). Spore-forming bacteria like *Sporosarcina pasteurii* and *Bacillus* spp.—many also electroactive—are resilient in harsh environments and effective in CO₂ sequestration and soil restoration (Omoregie et al., 2025; Shivaprakash and Burns, 2025). However, the applicability of electroactive microorganism (EAM)-based approaches across diverse climate zones and agricultural production systems need to be further explored. Environmental factors such as soil moisture regime, temperature, redox stratification, and organic matter availability—which differ markedly between irrigated systems, rain-fed agriculture, controlled-environment facilities, and extreme climates—strongly influence extracellular electron transfer and the stability of electroactive consortia (Sakai et al., 2022; Chen et al., 2023). While EAM-based systems are likely to be most robust under conditions that maintain persistent redox gradients, such as wetlands, irrigated soils, or greenhouse and vertical farming systems, their performance under highly variable or arid conditions remains uncertain (Garbini et al., 2023). Addressing these limitations will require systematic, comparative studies across climatic regions and management regimes, integrating soil physics, microbial ecology, and bioelectrochemical performance metrics. Incorporating climate and regional adaptability into future research agendas is therefore essential to ensure that EAM-enabled soil technologies evolve from conceptually promising tools into broadly applicable, climate-resilient components of sustainable agroecosystems.

Finally, EAMs contribute to soil stabilization, a crucial defense against erosion and land degradation. Ureolytic bacteria such as *Sporosarcina pasteurii* precipitate calcium carbonate, improving soil structure, water retention, and erosion resistance (Priyadarshi and Sharma, 2025). EAMs further enhance MICP efficiency by boosting urease activity (Fazelikia et al., 2023; Hemayati et al., 2023) and linking it to bioelectrochemical systems, where microbial fuel cell effluents stimulate calcifiers and support mineralization by taxa such as *Comamonadaceae*, *Arcobacter*, and *Aeromonas* (Hu et al., 2023; Liu et al., 2025). This integration reduces reliance on single strains and allows scalable stabilization strategies, though challenges remain, such as slower kinetics and uneven microbial growth compared to chemical approaches (Worley et al., 2024). Beyond soil stabilization, biocementation is now being extended into green material fabrication, including 3D-printed components for BES technologies, advancing circular and sustainable design (He et al., 2025; Horn et al., 2023; Dutto et al., 2024). Building on this vision, EAMs emerge not simply as soil inhabitants but as agents capable of reshaping agroecosystems into climate-smart, self-regulating systems.

## Synthesis and future vision: engineering climate-smart agroecosystems

This review highlights that EAMs might represent a turning point in how we actively manage soils—introducing new tools and approaches to guide and enhance soil functions. Channeling their capacity to couple energy transfer with nutrient cycling, they offer new ways to rebuild fertility, regenerate soil structure, and transform waste into valuable resources. Their ability to stabilize soils, detoxify pollutants, and support plant health positions them as versatile tools for creating resilient agroecosystems. Microorganisms, including EAMs, transform soils into intelligent, self-regulating systems that link biology with technology. It is, however, crucial to temper this vision with a realistic assessment of technological readiness. Current applications remain largely confined to proof-of-concept and pilot-scale studies. The inherent complexity of soil ecosystems, the challenge of maintaining engineered consortia *in situ*, and the current low power density of field-scale bioelectrochemical systems are significant hurdles to immediate widespread adoption. Therefore, the path forward requires a dedicated research agenda focused on materials science for durable electrodes, systems ecology for consortium integration, and techno-economic analyzes to validate feasibility. This balanced perspective acknowledges the transformative potential of EAMs while clearly framing the current stage of development and the necessary steps to advance from concept to reliable field application.

The future challenge is to translate these opportunities into practice at different scales, ensuring they are accessible to farmers and adaptable to diverse environments. This will require designing microbial communities suited to local soils, integrating bioelectrochemical platforms into farming systems, and aligning policies to support proactive soil management. With these steps, electroactive microorganisms can help engineer climate-smart agroecosystems—capable of sustaining productivity while enhancing ecological resilience.
